# Knowledge, Attitude, and Practices (KAP) About Vaccines Among Students in the Health Sciences Faculties in Kuwait

**DOI:** 10.3390/vaccines13121193

**Published:** 2025-11-25

**Authors:** Zahra K. Alsairafi, Abdallah Y. Naser, Abdullah N. Hasan, Ahmad Taqi, Mazen Ali, Sara Alsarraf

**Affiliations:** 1 Department of Pharmacy Practice, Kuwait University, Kuwait City 12037, Kuwait; zahra.alsairafi@ku.edu.kw (Z.K.A.); s2191125363@ku.edu.kw (M.A.); s2171120174@ku.edu.kw (S.A.); 2 Department of Applied Pharmaceutical Sciences and Clinical Pharmacy, Faculty of Pharmacy, Isra University, Amman 11622, Jordan; abdallah.naser@iu.edu.jo; 3 Mubarak Hospital, Ministry of Health, Kuwait City 47070, Kuwait; abd.nadar99@gmail.com

**Keywords:** Kuwait, health sciences students, vaccines, hesitancy, knowledge, attitude, practice

## Abstract

**Background:** Vaccination remains one of the most effective public health interventions, yet hesitancy persists even among healthcare students who aid in promoting immunization. Understanding students’ perspective plays a crucial role in designing targeted educational interventions. The purpose of this study was to evaluate knowledge, attitude, and practices of healthcare students (HSCs) in Kuwait about vaccines. **Methods:** A quantitative, cross-sectional study was conducted between August and October 2024. A validated 21-item questionnaire was used to assess vaccine-related knowledge, attitude, and practices, along with demographic data. Descriptive statistics and binary logistic regression were used to identify predictors of higher knowledge and positive attitude. **Results:** A total of 351 students participated (mean age 23.0 ± 2.4 years; 90.6% female). The mean knowledge score was 3.9/7 (55.7%), indicating moderate knowledge, with misconceptions noted regarding benefits of post-infection vaccination and extra vaccine doses. The mean attitude score was 3.6/6 (60%), indicating moderately positive attitude, yet safety concerns, particularly about long-term effect, were common (59.3%). Nearly half (45.9%) delayed vaccination until mandatory. Vaccine uptake was highest for COVID-19 (92.3%), followed by hepatitis B (73.8%). Older age, male gender, and being a medical student predicted higher knowledge (*p* = 0.011), while older age and being in later study years predicted more positive attitude (*p* = 0.032). **Conclusions:** HSCs demonstrated moderate knowledge and attitude toward vaccines, with significant hesitancy driven by safety concerns despite high eventual uptake. Early targeted curricular interventions addressing vaccine safety evidence, benefits of timely immunization, and professional responsibility are warranted to improve confidence and proactive vaccine acceptance among future healthcare professionals (HCPs).

## 1. Introduction

Vaccines have been used effectively to control many infectious diseases such as rubella, diphtheria, and polio and lower their mortality and morbidity rates [1]. However, as with many revolutionary ideas, the development and use of vaccines have been met with hesitancy and skepticism since that time [2,3]. The issue of vaccine hesitancy has persisted until this day and is still a challenge faced by public health. This has led the World Health Organization (WHO) to list vaccine hesitancy as one of the top ten global health threats in 2019, affecting both individual and public health [4]. Vaccine hesitancy is defined as a delay in acceptance or refusal of vaccines despite the availability of vaccination services. The refusal of vaccines increases the chance of being infected and the severity of the disease in individuals. Moreover, for vaccines to be effective on the societal level, herd immunity must be achieved [5]. Due to safety concerns, the challenge of vaccine hesitancy on public health has been made clear by the most recent pandemic caused by the SARS-CoV-2 virus (COVID-19), especially in countries in Europe and the Middle East [6,7,8]. This issue was particularly relevant in Kuwait which had the lowest acceptance rates worldwide (23.6%) for the COVID-19 vaccine, before it was available, forcing the government to take rigorous actions such as the country lockdown and the transition of education online after a period of discontinuation [9,10,11]. Such disruption in the country had a negative impact on both healthcare providers (HCPs) and students. Studies during the pandemic reveled high life stress and high depression and anxiety rates among HCPs and students of the health science centers (HSCSs) of Kuwait university (KU) [12,13]. The HSC of KU includes four faculties: medicine, dentistry, pharmacy, and allied health sciences. HSCSs are obliged to take certain vaccines such as hepatitis B, meningococcal, pneumococcal, flu, and COVID-19 during their clinical years, when they practice their knowledge in hospitals and polyclinics. This is to reduce the risk of infection among students and for the health and safety of patients.

However, vaccine hesitancy had an evident impact on HSCSs, where some of them prefer not to take the vaccines for some reasons and they start their clinical training without being vaccinated. During the pandemic of COVID-19, like the general population, some HSCSs demonstrated vaccine hesitancy due to concerns of its side effects and the misinformation from social media [14]. This prompted the government to release enforcing policies indicating that vaccination was crucial to return to on-site learning, clinical placements, and other social activities. Through this, uptake rates of COVID-19 vaccine increased among the public, including HCPs and HSCSs [14,15]. In 2023, a study reported high vaccine hesitancy and rejection rates for human papilloma virus (HPV) among female students in KU [16]. Another study in the Middle East reported considerably high influenza vaccine hesitancy rates for children among their parents [5]. Surprisingly, in that study, vaccine hesitancy was more prevalent in high-income countries such as Kuwait and among mothers with higher educational levels. However, in 2021, there was a case of one pharmacy student in KU being infected with meningitis during his clinical rotation, in which he was isolated and his study was disrupted. In addition, there were many cases of students being infected with flu, COVID-19, and pneumonia during their clinical rotations, by which their studies were temporarily interrupted. Unfortunately, to date there are no data determining the rates of infections among HSCSs. Due to the importance and effectiveness of vaccines, understanding hesitancy towards immunization especially in the healthcare field is essential. This is particularly important in Kuwait because of the low acceptance of vaccines, and the lack of information and research about this topic. In addition, in the future, the HSCSs can serve as a crucial source for scientifically validated information about vaccines influencing their uptake among the community. Therefore, this study aims to obtain knowledge, attitude, and practices (KAP) of HSCSs regarding vaccines to propose recommendations targeting vaccine-hesitant groups to improve vaccines uptake rates, reduce risk and spread of infectious diseases, and avoid study disruption for HSCSs.

## 2. Materials and Methods

### 2.1. Study Area and Design

Kuwait, a country in the Middle East, covers an area of 17,820 km^2^ and has an estimated population of 4,960,433 as of 2024 [9]. A quantitative, prospective, and cross-sectional study was performed to assess the knowledge, attitude, and practices of students in the HSC towards vaccines. It was conducted between August and October 2024. Ethical approval for this study was obtained from the Human Ethical Committee, HSC, Kuwait University (VDR/EC/569).

### 2.2. Study Population

The sample size was calculated following the WHO guidelines for the minimum required sample size in a prevalence study. With a 95% confidence interval, a standard deviation of 0.5, and a 5% margin of error, the necessary sample size was determined to be 289 participants. Eligible participants were invited to join the study through a convenience sampling method. Data collection was conducted using an online questionnaire administered via various social media platforms, such as WhatsApp, as well as self-administered questionnaires distributed to students at the HSC. To enhance outreach, the researcher utilized the official Twitter account of the HSC at Kuwait university for invitations. Additionally, a staff member from each faculty contacted students on their contact list through WhatsApp messages or e-mail.

The inclusion criteria for the study required participants to be students enrolled in health-related programs, such as medicine, dentistry, pharmacy, or allied health sciences, at their clinical years, e.g., year 3 and above. At the beginning of the survey, the study objectives were clearly outlined, along with assurances of confidentiality and anonymity. A reminder message was sent to all eligible participants before the data collection period concluded. Participation in the study was entirely voluntary, and all participants provided written consent.

### 2.3. Study Questionnaire

Previously used questionnaires by Lucia et al. ([17]), Naser et al. ([6]), and Shawqi et al. ([18]) were reviewed, comprised and adapted, and used in this study. The final version of the questionnaire includes 21 questions divided into three sections. Section 1 includes eight questions (K1–K8) to obtain knowledge of students in the HSC regarding vaccines. Questions 1–7 were answered on a yes/no basis with an additional “I don’t know” option. Question 8 was regarding the source of information about vaccines with “a-g” options listing different sources and an additional “others: specify” option. Section 2 includes eight questions (A1–A8) to obtain attitude of students in the HSC regarding vaccines. Questions 1 and 2 were about concerns from receiving vaccines, with “a-e” and “a-d” options, respectively. Questions 3–8 were answered on a yes/no basis with an additional “I don’t know” option. Section 3 includes five questions (P1–P5) to obtain practices of students in the HSC regarding vaccines. Questions 1 and 2 were about the taken vaccines and time of taking them, with “a-e” and “a-d” options, respectively. Question 3 was about whether the taken vaccine/s was by force or deliberately, with “yes”, “no”, and “I don’t know” options. Questions 4 and 5 were answered on a basis of 6-point Likert scale (ranging from Always–Not Applicable) and 5-point Likert scale (ranging from Always–Never), respectively. Question 4 was about applying the basic principles of infection control in clinical settings, while question 5 was regarding promoting vaccines to the public in the future.

In addition, demographic information was collected from the participants, such as age, gender, nationality, faculty, and year of study. All participants were asked whether they have been infected with one or more of the following viruses: hepatitis B, meningococcal, pneumococcal, flu, or COVID-19. The questionnaire was pre-tested for content, design, readability, and comprehension on five students, and minor modifications were included.

### 2.4. Data Analysis

Descriptive statistics were used to describe participants’ demographics. Continuous data were to be reported as mean (standard deviation (SD)). Categorical data were reported as percentages. Binary logistic regression was used to identify predictors of higher knowledge score and positive attitude towards vaccines and was presented as odds ratios (ORs) with 95% CIs. Logistic regression models used the mean knowledge score for the study sample (3.9) and the mean attitude score (3.6) as cut-off points to define the dummy variables for the regression models. A two-sided *p* < 0.05 was considered statistically significant. The statistical analyses were carried out using the Statistical Package for Social Sciences (IBM Corp., Armonk, NY, USA, SPSS Statistics for Windows, version 29).

## 3. Results

### 3.1. Demographics

The total number of participants was 420. There were 69 non-responders, which represented 19.6% of the total; thus, the final population included in this study was 351, which is a representative sample size according to our power calculations, with a predictive power of more than 80%. The majority of the participants, 90.6% (*n* = 318), were females, while 89.7% (*n* = 314) were Kuwaitis. Approximately half of the participants, 49.3% (*n* = 173), live in Hawalli and Al-Asimah governorate. Over half of the participants, 55.6% (*n* = 195), were pharmacy students. Most of participants were in year 5 of study, 43.9% (*n* = 154), followed by year 3, 27.4% (*n* = 96). Nearly half, 45% (*n* = 158), of the students were from high family income of more than KWD 2000, followed by medium family income of 40.5% (*n* = 142). Flu, 72.1% (*n* = 253), and COVID-19, 55% (*n* = 193), were the infections most contracted by students (Table 1).

### 3.2. Knowledge Regarding (Hepatitis B–Meningococcal–Pneumococcal–Flu–COVID-19) Vaccines

The mean knowledge score for the study’s sample was 3.9 (SD: 1.3) out of 7, which represents 55.7% of the maximum score and reflects a moderate level of knowledge. Data regarding knowledge about the significance of receiving more than a dose of certain vaccines showed that nearly half of participants, 53% (n = 186), did not believe in its efficacy. Most participants, 68.4% (240), believed that they can get infected with a pathogen by receiving its vaccine while 67.2% (n = 236) believed that they can be infected and spread the infection of the pathogen even if they already took its vaccine. Most of participants, 67.8% (n = 238), reported that vaccines would not negatively affect their immune system. Majority of participants, 81.8% (n = 287), indicated that infections could be spread among people even when the infected patient has no symptoms, while 73.8% (=259) determined that infections could lead to severe cases even among healthy young individuals. Data indicating main sources about vaccines among students showed that university education was the main source for 76.6% (n = 269) of the participants, followed by social media with a percentage of 54.1% (n = 190) (Table 2).

### 3.3. Attitude Regarding (Hepatitis B–Meningococcal–Pneumococcal–Flu–COVID-19) Vaccines

The mean attitude score for the study sample was 3.6 (SD: 1.6) out of 6, which represents 60.0% of the maximum score and reflects a moderately positive attitude towards vaccination. Majority of participants, 59.3% (n = 208), were concerned about safety and long-term side effects of vaccines; half of them, 51.3% (n = 180), showed their concerns about short-term side effects of vaccines, while most participants, 43% (n = 151), had concerns regarding the efficacy of vaccines. Receiving multi-doses of vaccines was the biggest issue concerning 51.9% (n = 182) of the participants. Regarding the necessity of learning about vaccine side effects, almost all the participants, 95.7% (n = 336), responded positively. Most participants, 67% (n = 235), agreed with the compulsory requirement of taking vaccines before their placement courses. Concerning receiving vaccines more than necessary as healthcare students, most participants, 56.7% (n = 199), responded positively. However, half of participants, 49.6% (n = 174), responded that they would still take vaccines even if it was not required from their university. Around one-third of the participants, 35% (n = 123), do not trust the information about vaccines provided by health institutions such as the MOH. A quarter of the sample, 26.2% (n = 92), believed that vaccines are prompted for financial gain or conspiracy (Table 3).

### 3.4. Practices Regarding (Hepatitis B–Meningococcal–Flu–COVID-19–Pneumococcal) Vaccines

Concerning the type of vaccine most taken by the participants, almost all the students, 92.3% (n = 234), received the COVID-19 vaccine, followed by hepatitis B vaccine, taken by the majority of the participants, 73.8% (n = 259). With regard to delaying the vaccine until it became a prerequisite to the placement course, roughly half of participants, 45.9% (n = 161), responded positively. With reference to applying basic principles of infection control in clinical settings, majority of participants, 68.7% and 59.3%, would always maintain hand hygiene, and safe handling and disposal of sharps and chemical waste, respectively. Regarding blood and bodily fluids and contaminating equipment, nearly half of participants, 53.6% and 49.9%, responded that they would always manage blood and fluids and decontaminating equipment, respectively. About promoting vaccines to patients in the future, more than half of the participants, 52.4% (n = 184), would always and often advise people to take vaccines (Table 4).

### 3.5. Predictors of Students’ Knowledge and Positive Attitude

Table 5 below shows the findings of binary logistic regression analysis which identified predictors of students’ knowledge and positive attitude. Older students (aged 23 years and older), males, and medicine students were more likely to be knowledgeable of vaccines compared to others (*p* = 0.032). Older students (aged 23 years and older) and those at their fifth and seventh years of study were more likely to have a positive attitude towards vaccines compared to others (*p* = 0.011).

## 4. Discussion

The present study assessed knowledge, attitude, and practices regarding vaccines among undergraduates in the HSC faculties in Kuwait. To our knowledge, this is the first study that addresses the hesitancy to accept vaccines among healthcare students covering several infectious diseases. In 2021, there were four studies that reported vaccine hesitancy rates for COVID-19 infection only among the public [9,10,11,14]. In 2023, another study addressed the vaccine hesitancy problem among female KU students for HPV infection only [16]. Overall, the participants in the current study had moderate knowledge regarding vaccines. However, there was a lack of knowledge regarding the benefits of receiving vaccines for a previously contracted infection, and there was the misconception that additional vaccine doses provide better protection against pathogens. The most common source of vaccine information, used by students, were local university education and social media. In addition, the participants had a moderately positive attitude towards vaccines. Most of the participants were concerned by the safety of vaccines, particularly short- and long-term side effects. Moreover, around half of the participants reported that they delayed receiving vaccines until it became mandatory, and some students would prefer not to receive any vaccine if it was optional. Regarding promoting vaccines to patients in the future, nearly half of the participants would usually advise patients to get vaccinated. Most of the participants reported following basic principles of infection control in clinical settings. These results are necessary, as it is the first study addressing hesitance to several vaccines among HSCSs in Kuwait, to allow for comparisons with similar studies in the Middle East and worldwide. Furthermore, such results may assist health authorities and KU in designing appropriate strategies that improve students’ willingness to vaccinate.

In the current study, there were important parallels when compared to other studies in Saudi Arabia, Qatar, and the United Arab Emirates (UAE) [19,20,21]. In those studies, suspicion about vaccines declined with academic advancement. For example, in Saudi Arabia, about one-third of students had negative attitudes towards vaccines, which was more common among pre-clinical students compared to clinical students. These results mirror findings of the current study where positive attitudes towards vaccination are more likely in older students and those who are in their fifth and seventh years of study, where students usually experience more clinical rotations. These similarities may highlight the need for curricular interventions targeting early-year students across the Gulf region. Regarding vaccine acceptance and preparedness to take vaccines, a quarter of the participants in Saudi Arabia would not take the COVID-19 vaccine if it is not required by their university. The acceptance rate for the COVID-19 vaccine among students in the UAE was 56.3% [21]. Similarly, in Kuwait, half of the HSCSs would not receive any vaccine unless mandated, and they often delayed vaccination until it became compulsory. On the other hand, students in Qatar showed their willingness to be vaccinated although they showed significant concerns about the vaccines’ safety and side effects. This could be related to the students’ confidence in the information provided by their government, as students in Qatar showed more confidence towards information provided from their Ministry of Health (MOH) than students in Kuwait and Saudi Arabia, respectively [19,20].

In the Middle East, several studies showed similar results to the current study in terms of showing a moderately positive attitude towards vaccines, and concerns regarding vaccines short and long-term safety [18,22,23,24,25]. For example, in Egypt, 43% of students had intermediate knowledge regarding vaccines, while 44.9% showed high concerns regarding their safety, which was a major reason for vaccination hesitancy among the students [18,22]. The current study also found that more than half of the students had a positive perception towards vaccines, but vaccination hesitancy persisted due to safety concerns. Concerns about possible side effects were also reported previously in Kuwait among the public for the COVID-19 vaccine [9,10,11,14]. This finding highlights a degree of ambivalence, as although students support vaccination in principle, strong concerns about safety and necessity continue to influence their decisions about receiving vaccines. This hesitancy, however, was less prevalent in participants who had clinical exposure, which is consistent with the current findings. This could be most likely attributed to witnessing the effects of vaccination in clinical rotations. In Morocco, vaccine uptake rates were more prevalent among students who perceived the efficacy of the vaccine and less prevalent among those who had higher concerns about vaccine safety [25]. Identifying interventions that alter students’ beliefs about vaccines could be a critical factor that should be targeted for HSCSs in Kuwait. Regarding vaccination uptake, the HSCSs in Kuwait had higher uptake rates especially for COVID-19, hepatitis B, and influenza vaccines than students in Egypt and Jordan [18,22,23,24]. This may be attributed to vaccination being a pre-placement requirement for students in Kuwait. Similarly, in Morocco and Jordan, around one-third of the participants showed their willingness to be vaccinated against COVID-19 [24,25]. However, most students in Jordan reported negative attitudes towards the COVID-19 vaccine and they rely on social media for information [24].

Globally, the experiences of medical and pharmacy students have been diverse, yet several consistent themes align with the findings of the current study. The HSCSs in Kuwait had higher knowledge regarding vaccines when compared to students in Brazil and Nigeria [26,27]. In Brazil, 64.2% of students lack awareness about the severity and frequency of vaccine-preventable infectious diseases. Accordingly, only few students received influenza vaccine [26]. However, in the USA, Romania, Italy, and Germany, students reported higher levels of knowledge and more positive attitudes towards vaccines [17,28,29,30,31]. In Italy, around 87.3% of participants supported mandatory vaccination to HCPs and more than half of them indicated they would recommend vaccines to the general population [31]. In the USA, around 77% of participants reported their willingness to receive the COVID-19 vaccine upon its Food and Drug Administration’s (FDA) approval [17,28]. Notably, participants in those studies reported national vaccination campaigns and scientific journals as their primary sources of vaccine-related information, whereas social media was the least frequently cited source. In contrast, in the current study, social media represented the second-most reported source of vaccine-related information. Therefore, the negative influence of social media and its role in spreading vaccine-related misinformation could contribute to the low vaccine perception among HSCSs in Kuwait. During the pandemic of COVID-19, some physicians posted videos on social media against receiving the vaccine, which contributed to the hesitancy rates among the students. Hence, to improve students’ confidence in health authorities in Kuwait, more supervision or applying strict disciplines for those who distribute misinformation on social media and provide inconsistent facts to that released from the MOH [32].

Moreover, the HSCSs in Kuwait highly expressed the belief that they were subjected to more vaccines than necessary, which may suggest that they perceive vaccination primarily as a personal health benefit rather than a collective responsibility. By contrast, students in Italy viewed vaccination as a moral obligation towards their society. However, despite the differences in their attitudes, HSCSs showed similar vaccine uptake rates to those in Italy, which could be attributed to the mandatory vaccination policy in Kuwait university [30]. Regarding the confidence in the information provided by HCPs and concerns about vaccine side effects, HSCSs had higher trust levels than students in the USA but similar concerns regarding vaccine side effects [17,28]. Surprisingly, although about three-quarter of students in Germany believed in the proven effectiveness of vaccines, over 80% of them had never had influenza vaccine records on their vaccination cards. This could be attributed to the students’ concerns (30%) about the safety of vaccines [31]. However, differences in immunization education between Middle Eastern countries and American and European countries should be taken into consideration. Vaccination constitutes a minor part of the KU curriculum that is discussed under broader topics such as the immune system, while it is integrated into more academic disciplines in other countries [33].

### 4.1. Strengths and Limitations

This study has several strengths and limitations. A key strength of this study is its comprehensive assessment of vaccine-related knowledge, attitudes, and practices among a diverse cohort of HSCs representing multiple faculties, including medicine, pharmacy, dentistry, and allied health. The use of validated questionnaires and robust statistical analyses, including logistic regression, allowed for the identification of predictors of both knowledge and attitudes, thereby enhancing the interpretability and applicability of the findings. Moreover, the relatively high response rate supports the representativeness of the sample within Kuwait university’s HSC.

However, the cross-sectional design method prevents the establishment of causal relationships between demographic variables and KAP outcomes. The sample was predominantly female and Kuwaiti, which may limit the generalizability of the findings to other populations or institutions within the Gulf region. Additionally, the reliance on self-reported data might have introduced recall bias, as participants may have inaccurately reported their vaccination history or practices. For future studies, longitudinal follow-up could be conducted to determine if current curricula naturally close the knowledge gap or if formal interventions are needed. Additionally, qualitative studies can be conducted to obtain a better understanding of students’ personal concerns about vaccine safety, perceived risk of disease, and influence of social media.

### 4.2. Implications for Policy and Practice

Vaccine hesitancy in the Gulf region continues to impact vaccine uptake rates and the spread of infectious diseases. This calls for multi-element interventions in public health communication and curriculum development to be undertaken. Policy makers should adapt approaches that address the determinants of vaccine hesitancy in the region such as fear of side effects, disbelief about vaccine effectiveness and lack of confidence in health authorities. The impact of religious beliefs and family and social norms in decision-making should also be taken into consideration. As HCPs are mentors to most people and healthcare students, they should be effectively trained to deliver culturally sensitive messages in collaboration with religious and community leaders to re-build the confidence in health authorities. Because social media continues to be a main source for information and misinformation in the region, dedicated strategies should be conducted to distribute evidence-based information that is consistent with information provided by the MOH of each country. As HCPs need to be continually trained, continuous training and monitoring for healthcare students is of a great significance. There are several approaches that could be adapted in KU by HCPs and academics to improve students’ willingness and understanding of vaccines; for example, motivational interviewing, reflective listening, and real-life consultations addressing the students’ concerns. In addition, increasing time and subjects discussing vaccination is important as it is often a minor part of curriculum [14,34,35].

## 5. Conclusions

Despite the moderate knowledge and moderately positive attitudes toward vaccines among HSCSs in this study, around half of them (45.9%) were hesitant about receiving vaccines and often delayed it until it became mandated. Furthermore, around 47.6% of HSCSs will not usually promote vaccines to people when they become HCPs. Safety concerns, particularly about short- and long-term adverse effects (51.3% and 59.3%, respectively) and lack of confidence in the information provided by governmental health authorities (more than a quarter of the participants), were the most common drivers of students’ hesitancy. In addition, more than 40% of HSCSs reported their concerns regarding the usefulness and efficacy of vaccines (particularly multi-dose vaccines), and they think that they take them more than necessary. These findings address the need for the following: (1) Effective educational campaigns addressing the susceptibility and severity of infectious diseases among healthcare students, especially during their clinical rotations. (2) Targeted curriculum interventions focusing on the potential benefits of vaccines, safety evidence, and emphasizing the responsibility of healthcare students in promoting immunization as they will be involved in direct patient care in the future. (3) Educational interventions that address students’ concerns supported with clinical evidence to build their trust in governmental health authorities and improve their willingness toward vaccines. (4) Initiating guidelines that organize the distribution of health information via social media. For example, the MOH could propose guidelines that compel HCPs to obtain approval from the MOH if they are planning to share any health information through social media and apply penalty for those who fail to do so. Finally, qualitative research could be conducted in Kuwait to further investigate the factors that contribute to vaccine hesitancy among students, especially males, those in the medical field, and in their first years of study.

## Figures and Tables

**Table 1:** Participants’ demographic characteristics.

Variable	Frequency	Percentage
**Mean age (standard deviation)**	23.0 (2.4) years	
**Gender**		
Females	318	90.6%
**Nationality**		
Kuwaiti	314	89.7%
**Place of residence (governorate)**		
Ahmadi	43	12.3%
Al-asimah (Capital)	79	22.5%
Farawniya	65	18.5%
Hawali	94	26.8%
Jahra	35	10.0%
Mubarak Al-kabeer	35	10.0%
**Faculty**		
Pharmacy	195	55.6%
Allied Medicine	105	29.9%
Dentistry	27	7.7%
Medicine	22	6.3%
Public health	2	0.6%
**Current year of study**		
Year 3	96	27.4%
Year 4	60	17.1%
Year 5	154	43.9%
Year 6	14	4.0%
Year 7	27	7.7%
**Family income**		
KWD 500–1000	51	14.5%
KWD 1000–2000	142	40.5%
More than KWD 2000	158	45.0%
**What infection have you been infected with previously?** (Multiple choice question)		
Flu	253	72.1%
COVID-19	193	55.0%
Pneumococcal	8	2.3%
Hepatitis B	14	4.0%
Meningococcal	9	2.6%

KWD: Kuwaiti dinar.

**Table 2:** Participants’ percentage of corrects answers to knowledge items.

Knowledge Item	Frequency (Percentage)	
	Yes	No
**K1-**Generally, does receiving more doses of a certain vaccine give better protection against the infection (the more the merrier)?	165 (47%)	186 (53.0%)
**K2-**Can you get infected by a pathogen by receiving its vaccine?	111 (31.6%)	240 (68.4%)
**K3-**Can you get the infection and spread it to other people even if you already have taken its vaccine?	236 (67.2%)	115 (32.8%)
**K4-**Is it beneficial to take the vaccine even if you have been infected with its targeted pathogen previously?	196 (55.8%)	155 (44.2%)
**K5-**Can vaccines be dangerous by negatively affecting the immune system?	113 (32.2%)	238 (67.8%)
**K6-**Can infected individuals transmit the infection to others when symptoms are not present?	287 (81.8%)	64 (18.2%)
**K7-**As a healthy young individual, can getting an infection result in a severe case?	259 (73.8%)	92 (26.2%)
**K8-**What is/are your main source(s)of information about the vaccines? (Multiple answers question)	My university education	269 (76.6%)
	Social media	190 (54.1%)
	Ministry of Health	176 (50.1%)
	World Health Organization	146 (41.6%)
	Medical staff members	142 (40.5%)
	Friends and family	105 (29.9%)
	Events (e.g., Workshops, conferences, etc…)	69 (19.7%)

**Table 3:** Participants’ answers to attitude items.

Attitude Item	Frequency (Percentage)	
**A1-**Generally, do any of the following concern you when it comes to receiving vaccines? (Multiple answers question):	a. Vaccine short-term side-effects	180 (51.3%)
	b. Usefulness/efficacy of vaccines	151 (43.0%)
	c. Long-term side-effects and safety	208 (59.3%)
	d. Generally, no concerns	74 (21.1%)
**A2-**What type of vaccines concern you more?	a. Single-dose	22 (6.3%)
	b. Multi-dose	182 (51.9%)
	c. Number of doses makes no difference (both are the same)	147 (41.9%)
**A3-**Do you think it is essential to learn about vaccines and their side-effects for yourself and your future patients?	**Yes**	**No**
	336 (95.7%)	15 (4.3%)
**A4-**Do you agree with the University’s decision about the compulsory requirement of taking these vaccines before the placements?	235 (67.0%)	116 (33.0%)
**A5-**Do you think healthcare students get more vaccines than is necessary?	199 (56.7%)	152 (43.3%)
**A6-**If vaccines are not a requirement in your university, would you still take them?	174 (49.6%)	177 (50.4%)
**A7-**Do you trust the information about vaccines provided by the Government/Ministry of Health?	228 (65.0%)	123 (35.0%)
**A8-**Do you think vaccines are being promoted for reasons other than health and safety (e.g., financial gain or a conspiracy)?	92 (26.2%)	259 (73.8%)

**Table 4:** Participants’ answers to practice items.

Practice Item						Frequency (Percentage)
**P1-What vaccine(s) did you take? (Yes)**						
a. Hepatitis B						259 (73.8%)
b. Meningococcal						239 (68.1%)
c. Pneumococcal						219 (62.4%)
d. Flu						228 (65.0%)
e. COVID-19						324 (92.3%)
**P3-**Did you delay taking any vaccine until it was necessary or enforced on you?						(Yes) 161 (45.9%) (No) 190 (54.1%)
**P4-**How often do you apply these basic principles of infection control, in clinical settings, if applicable?						
	Always	Often	Sometimes	Rarely	Never	Not applicable
Hand hygiene (washing your hands OR rubbing with alcohol)	68.7%	21.7%	7.4%	1.4%	0.3%	0.6%
Avoiding touching your face with your hands before washing them	41.3%	34.5%	16.0%	6.6%	0.3%	1.4%
Using personal protective equipment (like wearing face masks and gloves)	28.5%	24.8%	27.9%	13.4%	3.1%	2.3%
Safe handling and disposal of sharps/chemical waste	59.3%	14.8%	8.8%	3.7%	1.1%	12.3%
Managing blood and bodily fluids	53.6%	12.3%	7.7%	4.0%	3.1%	19.4%
Decontaminating equipment	49.9%	0.3%	10.5%	5.4%	2.3%	16.0%
**P5-**As a future healthcare professional, how often will you promote/advise people around you to get vaccinated?	24.5%	27.9%	29.1%	10.0%	8.5%	0.0%

**Table 5:** Predictors of students’ knowledge and positive attitude.

Variable	Odds Ratio of Having Higher Knowledge Score (95% Confidence Interval)	*p*-Value	Odds Ratio of Having Positive Attitude Knowledge Score (95% Confidence Interval)	*p*-Value
**Age category**				
Less than 23 years (Reference category)	1.00			
23 years and older	2.0 (1.3–3.1)	0.002 **	2.3 (1.5–3.6)	<0.001
**Nationality**				
Non-Kuwaiti (Reference category)	1.00			
Kuwaiti	0.3 (0.1–0.8)	0.014 *	1.0 (0.5–2.1)	0.926
**Faculty**				
Pharmacy (Reference category)	1.00			
Allied Medicine	1.2 (0.7–1.9)	0.500	0.4 (0.2–0.6)	<0.001
Dentistry	1.5 (0.6–3.5)	0.339	0.5 (0.2–1.1)	0.102
Medicine	3.4 (1.1–10.4)	0.032 *	1.2 (0.5–3.0)	0.740
Public health	-		-	
**Current year of study**				
Year 3 (Reference category)	1.00			
Year 4	0.6 (0.3–1.2)	0.125	1.4 (0.7–2.7)	0.309
Year 5	0.9 (0.5–1.5)	0.664	2.5 (1.5–4.3)	<0.001
Year 6	3.6 (0.8–17.0)	0.106	2.5 (0.8–8.1)	0.120
Year 7	1.4 (0.6–3.6)	0.452	3.3 (1.3–8.3)	0.011 *
**Family income**				
KWD 500–1000 (Reference category)	1.00			
KWD 1000–2000	0.8 (0.4–1.6)	0.611	1.1 (0.9–2.1)	0.755
More than KWD 2000	0.7 (0.3–1.3)	0.222	1.5 (0.8–2.7)	0.251

KWD: Kuwaiti dinar, * *p* < 0.05; ** *p* < 0.01.

## Data Availability

The original contributions presented in this study are included in the article. Further inquiries can be directed to the corresponding author.
